# Protective Loop Ileostomy Closure Techniques: Comparison of Three Different Surgical Techniques

**DOI:** 10.7759/cureus.10977

**Published:** 2020-10-16

**Authors:** Mustafa F Celayir, Mert Tanal, Evren Besler, Hakan Koksal

**Affiliations:** 1 General Surgery, Sisli Hamidiye Etfal Training and Research Hospital, University of Health Sciences, Istanbul, TUR

**Keywords:** ileostomy closure, ileostomy reversal, hand-sewn anastomosis, stapled anastomosis

## Abstract

Objective

Anastomotic leaks can be very dangerous in colorectal cancers. Protective loop ileostomy is life-saving in low anterior rectal tumors to prevent pelvic sepsis. The aim of this study is to compare early morbidities for stapled, handsewn closure (end to end) or handsewn closure (anterior wall only) of loop ileostomy, and to further assess efficacy and safety for each technique.

Methods

Patients who underwent loop ileostomy closure from January 2014 and December 2019 retrospectively were analyzed. Multivariate logistic regression was used to determine the effect of the potential risk factors on the rate of each complication. The patients were divided into three groups based on the anastomoses. The first group included patients who had handsewn anterior closure; the second group included patients who had side-to-side anastomosis using linear stapler, and the third group included patients who had end-to-end handsewn anastomosis. The primary endpoint of the study was the postoperative 30 days. IBM Statistical Package for the Social Sciences (SPSS), version 22.0 (SPSS Inc., Chicago, IL) was used for statistical analysis.

Results

A total of 198 patients underwent reversal. There was a statistical difference between the handsewn anterior wall and stapler anastomosis in terms of postoperative ileus and wound infection. The handsewn group was superior to anastomosis with stapler (p: 0.027 and p: 0.042, respectively). A statistical difference was found between handsewn anterior wall closure and handsewn end-to-end anastomosis in terms of postoperative ileus, wound infection, and postoperative hospital stay (p: 0.013, p: 0.037, and p: 0.046, respectively). When stapled anastomosis and handsewn end-to-end anastomosis techniques were compared, a statistical difference was found in terms of postoperative ileus risk (p: 0.043), but no significant difference was found in terms of postoperative wound infection and hospital stay.

Conclusions

There was no significant difference in the rate of anastomotic leakage between the handsewn and stapled techniques. The rate of small-bowel obstruction was higher in the handsewn group. As a result, in this study, it was revealed that the handsewn anterior wall closure technique is the best among all ileostomy closure techniques.

## Introduction

Colorectal cancers are the second most common cancer in cancer-related mortalities in Europe, and surgery is still the most effective treatment method [1]. Its treatment is based on tumor localisation, its stage, and spread; chemotherapy, radiotherapy and surgical treatments are related to each other [1,2]. The most fatal and frightening complication in rectal cancer surgery is anastomotic leakage [2,3]. In low-anterior and very low rectal cancers, the rate of anastomotic leakage is 3%-21% and the rate of mortality after leakage can be up to 22% [3]. Forming a protective loop ileostomy is recommended especially in low rectal tumors to prevent pelvic sepsis that may occur due to anastomotic leakage [3,4]. Loop ileostomies are closed in secondary operations and the postoperative complaints and morbidity of the patient change based on the closing technique [4]. The aim of this study was to evaluate the patients with diverting loop ileostomy in terms of the ileostomy closing techniques and to compare their morbidities.

## Materials and methods

The study included 198 adult patients who underwent ileostomy closure operation between January 2014 and December 2019. The underlying diseases, pre- and post-operative tests, and all radiological imagings of the patients were collected retrospectively. Pre-operative findings and applied surgical techniques were obtained from surgery records. The patients were divided into three groups based on the anastomotic techniques: The first group included patients who had handsewn anterior wall closure; the second group included patients who had side-to-side anastomosis using linear stapler; the third group included patients who had end-to-end manual anastomosis. The patients who had a history of colorectal additional operations and who mostly had an emergency Hartmann procedure and then their colostomy was closed and converted to an ileostomy were not included in the study. The patients with an incisional hernia and/or parastomal hernia were also excluded from the study. There were 64 patients in the first group, 78 in the second group, and 56 in the third group. The ileostomy closure was performed approximately in the 5th-6th months in all patients. The patients whose ileostomies were closed early due to high output and electrolyte imbalance were included in the study. Before the take down operations, all patients underwent imaging, and the possibility of anastomotic stenosis or recurrence was eliminated with colonoscopy.

In all patients, after passing through the skin-subcutaneous ileostomy line with a fish-mouth oblique incision, the abdomen was entered by passing through the posterior and anterior rectus fascia separately and then peritoneum. Along with the mesentery, the ileostomy was separated from the surrounding adhesions often with mostly sharp and also blunt dissections by preserving the afferent and efferent loops. Then, one of the three techniques for anastomosis was performed by the patient’s surgeon according to their preference. As the standard, in all patients, no subcutaneous suture was placed, ileal mesenteric planes were carefully handled, and no injuries were caused in the previous operation. As the standard, no intestinal fascia was detected while forming an ileostomy on the patients during their previous operation. Additionally, the mean postoperative hospital stay duration was planned as four-six days and the patients’ transition to enteral feeding was gradually arranged. Postoperative imaging methods were not applied routinely and were only applied in patients whose physical examination findings or laboratory tests continued to have acute phase reactant elevation.

In this study, three surgeons with over 10 years of experience in general surgery, and three techniques were evaluated retrospectively. The aim was to evaluate the factors that affect postoperative morbidity by eliminating the variable of three surgical techniques, surgical experience, and team factor. All surgeons used all three techniques randomly. There was no single specific method for each surgeon.

The primary endpoint of the study was the postoperative 30 days. For the postoperative morbidity follow-up, the results of both pre-operative laboratory and radiological tests and the tests made in the postoperative 30 days were included in the evaluation. Statistical Package for the Social Sciences (SPSS), version 22.0 (SPSS Inc., Chicago, IL) was used for statistical analysis; a p value under 0.05 wasconsidered significant in multivariate analyses.

## Results

Considering the demographic data of 198 patients included in the study, 101 patients were male and 97 patients were female. The mean age was 59.21 (±3.56) and the mean duration of postoperative hospital stay was 6.2±1.7 days (Table 1).

**Table 1:** Demographic and general clinical characteristics of the patients SD: standard deviation.

Specifications	
Patient's age, Year (mean±SD)	59.21±3.56
Sex, n (%)	
Male	101 (51)
Female	97 (49)
Additional disease n (%)	
Diabetes Mellitus	46 (23.2)
Chronic hypertension	48 (24.2)
Cardiac history and angiography	42 (21.2)
Length of Hospital Stay, day (mean±SD)	6.2±1.7

The patients were divided into three groups based on intestinal anastomosis: The first group included patients on whom anterior wall closure was performed by hand, whereas the second group included patients on whom side-to-side anastomosis was made using linear stapler; and the third group included patients on whom handsewn end-to-end anastomosis. There were 64 patients in the first group, 78 in the second group and 56 in the third group. There were no statistically significant differences between the patient groups that could contribute to the complication rates. All surgeons used all three techniques randomly. There was no single specific method for each surgeon (Table 2).

**Table 2:** Distribution of different closure techniques for each surgeon.

Patient groups	Surgeon 1	Surgeon 2	Surgeon 3
Handsewn anterior wall closure	28	22	21
Side-to-side with linear stapler	20	30	15
Handsewn end-to-end	16	26	20
Total	64	78	56

Considering the intergroup comparisons of postoperative ileus development, postoperative wound site infection frequency and the total duration of hospital stay, a statistical difference was found between the groups 1 and 2 in terms of postoperative ileus and wound site infection, and handsewn anterior wall closure was found to be superior to the side-to-side anastomosis with a stapler (respectively, p: 0.027 and p:0.042) (Table 3).

**Table 3:** Evaluation of the postoperative characteristics of patients with handsewn anterior closure (group 1) and side-by-side stapled anastomosis (group 2) Pearson Chi-Square Test  *p<0.05

	Group 1 (N:64)	Group 2 (N:78)	P
Postoperative ileus n (%)	1 (1.56)	6 (7.69)	0,027*
Postop wound infection n (%)	3 (4.69)	9 (11.54)	0,042*
Length of hospital stay, day (mean±SD)	5.3±1.3	5.9±1.2	0,273*

There were no significant differences between group 1 and group 3 in terms of postoperative hospital stay duration. In the comparisons between anterior closure by hand and end-to-end anastomosis by hand, a significant difference was found between the group 1 and group 3 in terms of postoperative ileus, wound site infection and postoperative hospital stay duration (respectively, p: 0.013, p: 0.037 and p:0.046) (Table 4).

**Table 4:** Evaluation of postoperative characteristics of patients who underwent handsewn anterior closure (group 1) and handsewn end-to-end anastomosis (group 3) Pearson Chi-Square Test  *p<0.05

	Group 1 (N:64)	Group 3 (N:56)	P
Postoperative ileus n (%)	1 (1.56)	11 (19.64)	0,013*
Postop. wound infection n (%)	3 (4.69)	10 (17.86)	0,037*
Length of hospital stay, day (mean ±SD)	5.3±1.3	6.4±1.8	0,046*

Considering the comparisons between the techniques of ileostomy closure by performing side-to-side anastomosis with a stapler and handsewn end-to-end anastomosis, a statistically significant difference was found between the group 2 and group 3 in terms of postoperative ileus risk (p: 0.043), but there were no significant differences in terms of postoperative wound site infection and duration of hospital stay (Table 5).

**Table 5:** Evaluation of the postoperative characteristics of patients who underwent side-by-side stapled anastomosis (group 2) and handsewn end-to-end anastomosis (group 3) Pearson Chi-Square Test  *p<0.05

	Group 2 (N:78)	Group 3 (N:56)	P
Postoperative ileus n (%)	6 (7.69)	11 (19.64)	0,043*
Postop wound infection n (%)	9 (11.54)	10 (17.86)	0,611
Length of hospital stay, day (mean±SD)	5.9±1.2	6.4±1.8	0,397

In addition to these data, postoperative anastomotic leakage was detected in two patient - one with a side-by-side anastomosis made with a stapler and one with a handsewn end-to-end anastomosis. These two patients were operated and a protective loop ileostomy was formed again.

## Discussion

Previous studies showed that the risk of anastomotic leakage, in anastomoses closer than 8 centimeters to the anal werge, in colorectal cancers may rise to 24% [3]. Therefore, temporary protective loop ileostomies have almost become a routine in surgeries to protect the anastomosis. The advantages of ileostomy over colostomy are that ileostomies are easier to open and close in terms of surgical technique, its edematous appearance is less and easier to work, and its complications such as prolapse and parastomal hernia are less common [5]. The mortality rate is low in ileostomy closures (0.4%), but the risk of morbidity and complications is substantially high (20%) [6,7].

Ileostomy closure operations are one of the abdominal operations where the dissection should be made in the most careful way, and the surgeon should always be careful and be prepared for a bowel injury. While bowel injuries induced by adhesions should be avoided in operations performed on the abdominal midline, the dissection should be made without damaging the afferent and efferent loops of ileostomy in operations performed in the shape of a fish-mouth around the ileostomy. In case of damage that may occur on these loops or mesentery, the anastomosis area to be made over the ileostomy is at risk, and additional resections might be performed. In our cases, our surgery teams made meticulous dissection, and mesentery damage did not occur.

Shortening and thickening of the mesentery feeding the afferent and efferent loops of ileostomy, bleeding from epigastric vessels in the rectus sheath, and atrophy-stenosis in the efferent loop are among common peroperative difficulties encountered during stoma closure [8]. Bleedings on the rectus sheath should be stopped, and the dissection should be made carefully on the mesentery feeding the efferent and afferent loops. Stenosis induced by edema formation on the efferent loop after anastomosis may cause postoperative intestinal obstruction [9]. In this study, especially among 34 patients who were recorded to have a narrow efferent loop in the operation reports as a peroperative finding, postoperative bowel motility of 11 patients was delayed and the patients’ length of hospital stays was prolonged.

Ileostomy closure operations are quite difficult in patients with obesity [10]. Morbidity increasing factors such as wound site infection and late-onset of postoperative bowel motility are more apparent in patients with obesity [11]. Especially the difficulty of providing adequate sight in patients with obesity, it is among the main factors that complicate the dissection in obese patients with an ileostomy [12]. The mean duration of postoperative hospital stays of 22 patients with body mass index (BMI) >30 was 6.2 days in this study. Wound site infection developed in six patients and the mean duration of hospital stay of these patients was 11.6 days.

Similar to postoperative mechanic intestinal obstruction, wound site infection is a common complication encountered after ileostomy closure operations [13]. Wound site infections, which increase morbidity and hospital stay and which is completed with the secondary recovery processes, are reported in the literature at the rate of 3%-40% and generally do not have a sole impact on mortality [14]. In our study, however, there were no significant differences between group 1 and group 2 in terms of postoperative hospital stay duration. In comparison, a significant difference was found between group 1 and group 2 in terms of postoperative ileus, wound site infection, and postoperative hospital stay duration (respectively, p: 0.013, p: 0.037, and p:0.046).

Previous studies have shown that the complication rate of the side-to-side anastomosis with stapler is lower compared to the handsewn end-to-end anastomosis [15]. In anastomosis with stapler, no resection was needed, no dissection in the intestinal mesentery was needed, the operation can be completed quickly, and the risk of postoperative bowel obstruction is low since adequate lumen width is provided [15,16].

The researchers compared the techniques of anterior closure by hand, end-to-end closure by hand, and side-to-side anastomosis with stapler and found that handsewn anterior wall closure is better than the other two techniques considering paired group comparisons. The superiority points of anterior wall closure by hand over side-to-side anastomosis with stapler are that there is no change in the functional bowel tissue and the passage direction, there is minimal contact with the ileum, and that this technique does not cause peroperative small bowel rotations, and these are considered as the factors that may explain the low risk of ileus development in anterior closure by hand. Similarly, ileostomy closure through a side-to-side anastomosis with stapler requires small bowel segments to be taken out more from the incision, and the duration of contact with subcutaneous fatty tissues prolongs. This increases the risk of postoperative wound site infection. There are studies on the duration of postoperative hospital stay in the literature, and some of these studies found significant differences between surgical techniques in terms of hospitalization duration while some studies found no significant differences similar to this study [17,18].

The necessity to completely separate and release mesentery tissues feeding afferent and efferent loops that come to ileostomy by forming the anastomosis with continuous sutures in the end-to-end anastomosis by hand technique is among the factors that may cause postoperative ileus [19,20]. The risk of ileus in the end-to-end anastomosis will be higher compared to a large anastomosis like side-to-side anastomosis with stapler since the diameter of the anastomosis formed with the handsewn end-to-end anastomosis will be smaller than the diameter of the ileal loop and even smaller than the afferent loop, and will be close to the efferent loop. The risk of ileus was found to be higher in the handsewn end-to-end closure compared to handsewn anterior wall closure technique where the diameter did not change and side-to-side anastomosis technique with stapler technique where anastomosis was large in this study.

## Conclusions

In conclusion, it has been revealed that the anterior closure anastomosis by hand technique was statistically superior over the side-to-side anastomosis with stapler and end-to-end anastomosis by hand techniques in terms of general postoperative morbidity, normalization in bowel motility, and duration of hospital stay; there were no differences between three techniques in terms of mortality. The important thing in protective loop ileostomy is to perform the dissection very carefully, avoid peroperative bowel injuries, and provide anastomosis to ensure the width to prevent stenosis.

## References

[1] McDermott FD, Heeney A, Kelly ME, Steele RJ, Carlson GL, Winter DC (2015). Systematic review of preoperative, intraoperative and postoperative risk factors for colorectal anastomotic leaks. Br J Surg.

[2] Jannasch O, Klinge T, Otto R (2015). Risk factors, short and long term outcome of anastomotic leaks in rectal cancer. Oncotarget.

[3] Phillips BR, Harris LJ, Maxwell PJ, Isenberg GA, Goldstein SD (2010). Anastomotic leak rate after low anterior resection for rectal cancer after chemoradiation therapy. Am Surg.

[4] Nicolau AE (2011). Temporary loop-ileostomy for distal anastomosis protection in colorectal resections [Article in Romanian]. Chirurgia (Bucur).

[5] Tilney HS, Sains PS, Lovegrove RE, Reese GE, Heriot AG, Tekkis PP (2007). Comparison of outcomes following ileostomy versus colostomy for defunctioning colorectal anastomoses. World J Surg.

[6] Penninckx F (2011). Anastomotic leakage: a disaster or a challenge with an impact on survival after rectal cancer surgery?. Colorectal Dis.

[7] Richards CH, Campbell V, Ho C, Hayes J, Elliott HT, Thompson‐Fawcett M (2012). Smoking is a major risk factor for anastomotic leak in patients undergoing low anterior resection. Colorectal Dis.

[8] Menahem B, Lubrano J, Vallois A, Alves A (2018). Early closure of defunctioning loop ileostomy: is it beneficial for the patient? A meta-analysis. World J Surg.

[9] Kelly-Schuette K, Wilkes A, Kyriakakis R, Ogilvie J (2020). Predictors of hernia after loop ileostomy closure: a single-center retrospective review. Int J Colorectal Dis.

[10] Saito Y, Takakura Y, Hinoi T, Egi H, Tashiro H, Ohdan H (2014). Body mass index as a predictor of postoperative complications in loop ileostomy closure after rectal resection in Japanese patients. Hiroshima J Med Sci.

[11] De Robles MS, Bakhtiar A, Young CJ (2019). Obesity is a significant risk factor for ileostomy site incisional hernia following reversal. ANZ J Surg.

[12] Bertelsen CA, Andreasen AH, Jorgensen T (2010). Anastomotic leakage after anterior resection for rectal cancer: risk factors. Colorectal Dis.

[13] Nunoo-Mensah JW, Chatterjee A, Khanwalkar D, Nasmyth DG (2004). Loop ileostomy: modification of technique. Surgeon.

[14] Chow A, Tilney HS, Paraskeva P, Jeyarajah S, Zacharakis E, Purkayastha S (2009). The morbidity surrounding reversal of protective loop ileostomies: a systematic review of 48 studies including 6,107 cases. Int J Colorectal Dis.

[15] Leung TTW, MacLean AR, Buie WD, Dixon E (2008). Comparison of stapled versus handsewn loop ileostomy closure: a meta-analysis. J Gastrointest Surg.

[16] Gong J, Guo Z, Li Y, Gu L, Zhu W, Li J, Li N (2013). Stapled vs hand suture closure of loop ileostomy: a meta-analysis. Colorectal Dis.

[17] Man VC, Choi HK, Law WL, Foo DC (2016). Morbidities after closure of ileostomy: analysis of risk factors. Int J Colorectal Dis.

[18] Baraza W, Wild J, Barber W, Brown S (2010). Postoperative management after loop ileostomy closure: are we keeping patients in hospital too long?. Ann R Coll Surg Engl.

[19] Mengual-Ballester M, García-Marín JA, Pellicer-Franco E, Guillén-Paredes MP, García-García ML, Cases-Baldó MJ, Aguayo-Albasini JL (2012). Protective ileostomy: complications and mortality associated with its closure. Rev Esp Enferm Dig.

[20] Löb S, Luetkens K, Krajinovic K, Wiegering A, Germer CT, Seyfried F (2018). Impact of surgical proficiency levels on postoperative morbidity: a single centre analysis of 558 ileostomy reversals. Int J Colorectal Dis.

